# Bottom-Up Assembly of Hydrogels from Bacteriophage and Au Nanoparticles: The Effect of Cis- and Trans-Acting Factors

**DOI:** 10.1371/journal.pone.0002242

**Published:** 2008-05-21

**Authors:** Glauco R. Souza, Esra Yonel-Gumruk, Davin Fan, Jeffrey Easley, Roberto Rangel, Liliana Guzman-Rojas, J. Houston Miller, Wadih Arap, Renata Pasqualini

**Affiliations:** 1 Department of Genitourinary Medical Oncology, The University of Texas M. D. Anderson Cancer Center, Houston, Texas, United States of America; 2 Department of Cancer Biology, The University of Texas M. D. Anderson Cancer Center, Houston, Texas, United States of America; 3 Department of Chemical Engineering, Massachusetts Institute of Technology, Boston, Massachusetts, United States of America; 4 Department of Chemistry, The George Washington University, Washington, D. C., United States of America; The University of Manchester, United Kingdom

## Abstract

Hydrogels have become a promising research focus because of their potential for biomedical application. Here we explore the long-range, electrostatic interactions by following the effect of trans-acting (pH) and cis-acting factors (peptide mutation) on the formation of Au-phage hydrogels. These bioinorganic hydrogels can be generated from the bottom-up assembly of Au nanoparticles (Au NP) with either native or mutant bacteriophage (phage) through electrostatic interaction of the phage pVIII major capsid proteins (pVIII). The cis-acting factor consists of a peptide extension displayed on the pVIII that mutates the phage. Our results show that pH can dictate the direct-assembly and stability of Au-phage hydrogels in spite of the differences between the native and the mutant pVIII. The first step in characterizing the interactions of Au NP with phage was to generate a molecular model that identified the charge distribution and structure of the native and mutant pVIII. This model indicated that the mutant peptide extension carried a higher positive charge relative to the native pVIII at all pHs. Next, by monitoring the Au-phage interaction by means of optical microscopy, elastic light scattering, fractal dimension analysis as well as Uv-vis and surface plasmon resonance spectroscopy, we show that the positive charge of the mutant peptide extension favors the opposite charge affinity between the phage and Au NP as the pH is decreased. These results show the versatility of this assembly method, where the stability of these hydrogels can be achieved by either adjusting the pH or by changing the composition of the phage pVIII without the need of phage display libraries.

## Introduction

Bottom-up nanoparticle assembly has recently been considered as a model for the development of enabling technologies in bioengineering and biomedical research [1], [2]. Tailoring nanoparticle architecture requires the challenge of reproducibly manipulating and controlling assembly at the molecular level. The integration of inorganic nanoparticles into biological systems, by means of synthetic biology fabrication, may offer solutions needed to overcome such tasks. Bioinorganic assembly, such as aggregates of nanoparticles and filamentous bacteriophage [3] (phage), have shown many potential applications, ranging from cancer cell targeting, bio-materials/electronics, to stem cell tissue engineering [1], [4], [5], [6], [7], [8], [9]. For large-scale fabrication of hybrid structures, one relies on controlling the various types of non-covalent interactions, long-range (i.e. electrostatic) and short-range (i.e. van der Waals) [8], [9], [10], [11], typically between nanoparticles and proteins, in which chemical and structural complexity makes elucidation of the assembly mechanism difficult to fully characterize.

**Figure 1:**
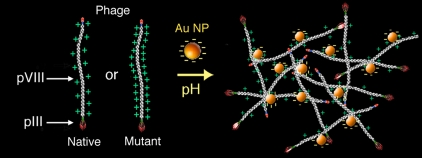
Scheme of Au-phage hydrogel assembly with native and mutant phage. The pVIII and pIII arrows point to the major (white region) and minor phage (red region) capsids, respectively (not drawn to scale).

Here we explore the long-range, electrostatic interactions by following the effect of pH on the assembly of Au-phage hydrogels. Hydrogels have become a promising research focus because of their potential broad biomedical application, including tissue engineering, wound healing, drug/gene delivery and stem cell manipulation. In our work, bioinorganic assemblies are generated from the cross-linking of Au nanoparticles with either native or mutant phage through electrostatic interaction of the pVIII major capsid proteins (pVIII). An alternative approach to the controlled assembly of nanoparticles onto phage has been presented in which peptide selection for metal binding is performed through display on the pIII minor capsid protein (pIII) followed by genetic manipulation of the phage's pVIII to display the selected peptide [4], [5]. Both the native pVIII and the library-selected amino acid sequences of the metal-binding peptides show a low frequency of the typical amino acids that have the highest affinity for metals [8], [9], such as histidines and cysteines [10], [11], [12]. Electrostatic interactions play a major role in the stabilization and functionality of bioinorganic molecules [10], [11], [13], [14] and one would expect that charge-mediated forces contribute to the building and stability of phage and Au NP assembly. The assembly and stability of the Au-phage hydrogels likely results from a complex interplay of ligand-exchange [15], long- and short-range interactions, which include electrostatic forces (long-range nature). Here, we focus on addressing the contribution of electrostatic interactions in the onset and stability of the hydrogel assembly as a function of phage major capsid composition. We show that pH conditions can direct hydrogel formation and stability by mediating the coupling of Au NP (citrate reduced) [13], [14] and the pVIII (Fig. 1). By adjusting the pH, differences between native or mutant major capsid can be offset assuring hydrogel assembly with either phage system.

## Results

### Phage charge computation

**Figure 2:**
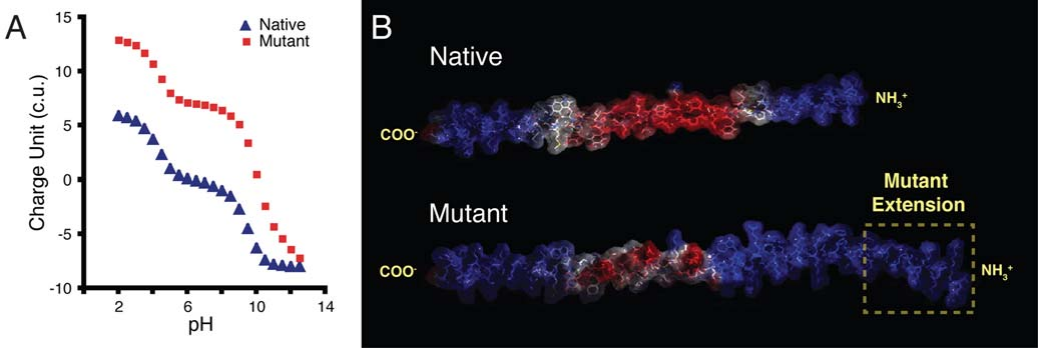
Calculations for native and mutant pVIII charge. (A) Theoretical calculation based on individual amino acid charge of the native and mutant pVIII protein as a function of pH. (B) pVIII structure generated using DeepView images of the native (top) and a mutant version (bottom) of the pVIII protein. The positively charged domains are shown in blue and negative ones are in red. Hydrophobic regions appear white. The native sequence was: NH_3_^+^-aegddpakaafdslqasateyigyawam vvvivgatigiklfkkfts-COO^−^ and the mutant one was NH_3_^+^-PRQIKIWFQNRRMKWKKPae gddpakaafdslqasateyigyawamvvvivgatigiklfkkfts-COO^−^ (capitalized residues and boxed region represent the mutant peptide extension).

Because many of the phage physical-chemical properties are defined by the structure and charge of the pVIII displayed peptides, the first step in characterizing the Au NP interaction with phage was to generate a molecular model which identified the charge distribution and structure of the native and mutant pVIII protein (Fig. 2A and B). The mutant phage were genetically modified [4], [5] to display a peptide extension of arbitrary sequence that carried a higher positive charge character relative to the native pVIII at all pHs (Fig. 2A). The graphical outcome of these calculations (with GRASP and DeepView software) shows the positive regions of the pVIII peptide in blue, the negative domains in red, and hydrophobic regions in white (Fig. 2B).

### pH dependence studies

**Figure 3:**
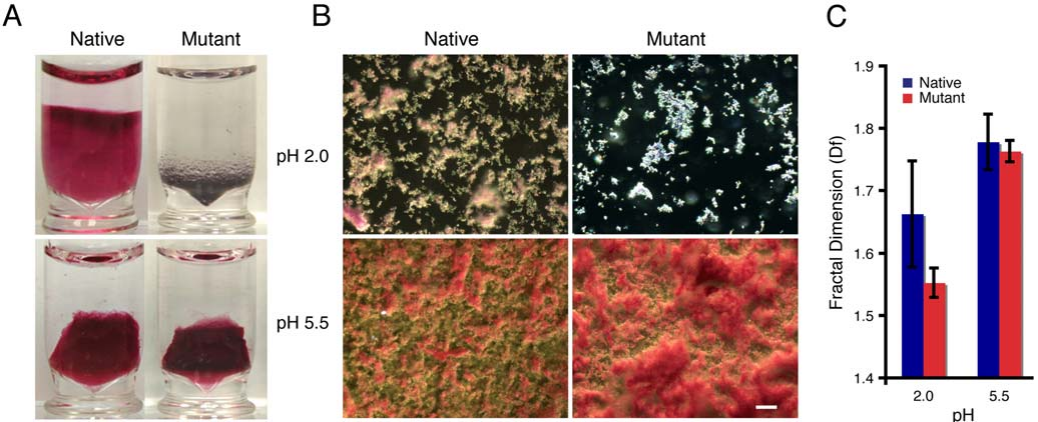
Effect of pH in the formation and structure of hydrogels assembled with native or mutant phage. (A) Vials with hydrogels prepared with native and mutant phage at pH 2.0 (top) and 5.5 (bottom). Au NP of 50±8 nm were prepared by citrate reduction. (B) Darkfield micrographs (elastic scattering) of hydrogels in (A) (constant light intensity). (Scale bar, 10 µm). (C) *Df* of structures in (B) determined with box counting *Df* analysis (ImageJ software).

**Figure 4:**
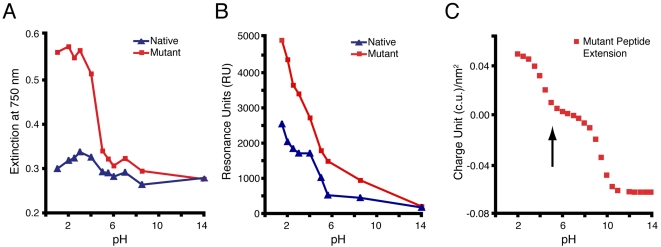
Differences in light extinction and SPR signal between the native and mutant Au-phage hydrogels as a function of pH. (A) Light extinction at 750 nm for hydrogels as a function of pH. (B) SPR measurements of phage interaction with citrate-treated Au surface vs. pH (buffer background signal subtracted from data). (C) Surface charge density calculation based on individual amino acid charge present on mutant peptide extension.

In order to characterize and compare the interactions of Au NP with either native or mutant phage, we evaluated the dependence of several experimental observables as a function of pH. We first identified the formation of Au-phage hydrogels with either native or mutant phage (Fig. 3A, suspended gel structures with red coloration). Then, darkfield microscopy (elastic scattering, Fig. 3B) and fractal dimension (*Df*) analysis (Fig. 3C) was applied to quantify the differences in microstructures of the hydrogel systems (Fig. 3B). The fractal dimension (*Df*) of a nanoparticle assembly is a quantitative measure of their structural organization [16], [17]. After this, we performed two separate pH titrations. In the first titration, we measured the Au-phage optical properties by means of Uv-vis spectroscopy (Uv-vis). When using Uv-vis, the Au NP-phage interactions (native and mutant) were detected by monitoring the shift in the plasmon resonance of the Au NP into the NIR (Fig. 4A). This red shift (indicated by the increase in 750 nm extinction) generally occurs when the distance between Au NP (*d*Au) is less than the average particle diameter (2*r*Au; *d*Au≤2*r*Au) [13], [14]. In the second titration, we used surface plasmon resonance (SPR) spectroscopy, where a Au surface is used to evaluate the Au-phage interaction without the presence of Au NP [11], [18]. Furthermore, the purpose of using SPR spectroscopy is to use a Au surface as a sensor and to mimic the Au NP-phage interaction (Fig. 4B).

## Discussion

### Phage charge computation

The higher positive charge distribution of the mutant pVIII (Fig. 2B) is in qualitative agreement with the charge estimated from the primary sequence of the peptide extension (Fig. 2A). The assembly and stability of hydrogels is due to the interplay of ligand exchange, long- and short-range interactions. For Au-phage networks, the interactions are convoluted by the adsorbed citrate layer on the particle surface, which is oriented with the six carboxylic oxygen atoms aligned with gold atoms of a 111 surface [19]. Chen et al. proposed that molecules containing carboxylic acid groups can displace citrate on gold surfaces through ligand exchange [15]. The molecular model images (Fig. 2B) show that many of these acids are oriented with the carboxylic acid groups pointed outwards, which suggests that formation and stability of Au-phage assembly for the native phage is a combination of citrate replacement coupled with electrostatic interaction between the positive residues on the pVIII and Au NP. In the mutant pVIII, more positive character is available in the exposed capsid surface, which should influence the phage interaction with negatively charged Au NP as a function of pH.

### pH dependence studies

All four experiments indicated a distinct pH dependence behavior between the native and mutant phage. This difference was visually detectable (Fig. 3A and B) as well as quantifiable from the *Df* analysis from the elastic scattering images (Fig. 3C). First, at pH 2.0 the mutant system failed to form hydrogel structures (Fig. 3A top right vial) indicated by the distinct light scattering behavior (white color instead of pink structures) and lower *Df* (1.50±0.07) than all other systems (Fig. 3C). Next, the pH titrations using either 750 nm extinction (with Au NP; Fig. 4A) or SPR spectroscopy (Au flat surface, absence of Au NP; Fig. 4B) measurements, underscored the assembly differences between the two systems. Both titrations showed a “threshold” behavior for the mutant system, where a sharp increase in extinction (Fig. 4A) and SPR (Fig. 4B) signal was observed for pHs below 5.0. Notably, this pH threshold (pH = 5.0) overlaps with the onset of the calculated net positive charge (pH < pI) of the mutant peptide extension (Fig. 4C and Fig. 2B, dashed box). The SPR measurements followed the same trend, with an increase in phage adsorption to the Au surface at a lower pH (pH≤5.0). At pH 2.0, the mutant phage SPR signal is twice as high (5,000 RU) as the one with the native phage (2,500 RU, Fig. 4B). This result supports the hypothesis that the predicted increase in positive charge of the mutant peptide extension as the pH is decreased (pH≤5; Fig. 4C) favors the opposite charge affinity between the phage and the Au NP. Furthermore, the increased phage adsorption observed with mutant phage system (Fig. 4B), seem to prevent hydrogel formation (Fig. 2A), indicated by the presence of the red shift in absorption from Au NP (Fig. 4A). One must emphasize that the Au NP are dispersed within the hydrogel structures (*d*Au>2*r*Au; Fig. 3A, top-left, bottom-left and bottom-right vials) [1], indicated by the low 750 nm extinction signals (Fig. 4A). Therefore, at pH 2.0, the high 750 nm extinction values of the mutant phage system, resulting from the shortening of the Au NP inter-particle distance, indicates an increase in Au NP density. This increased density likely contributes in preventing the formation of the water-suspended hydrogel assemblies (Fig. 3A, top right).

These results show that by tuning the pH, and therefore controlling phage surface charge, the direct-assembly of Au NP and phage can be induced into stable hydrogels in spite of the differences in the phage major capsid protein and without the need of phage display library screening. Furthermore, because of the significant structural and chemical differences in the environment in which the pIII and pVIII proteins are displayed, such pH dependence studies can provide additional insight into the interaction and assembly role of peptide motifs selected with pIII libraries within the more representative context of the pVIII environment. When considering the phage surface area (∼18,500 nm^2^, mostly made of pVIII) and capsid dimensions [4], [5], the location and structural environment of the minor capsid consist of 3 to 5 copies of the pIII displayed on an area (at one of the phage extremity; Fig. 1) of approximately 28 nm^2^, which translates to a peptide surface density of approximately 2–3 peptide/nm^2^. In contrast to the minor capsid, the pVIII mutant peptide extension are dispersed across the surface of the major capsid with approximately one mutant extension displayed for every 26 native copies of pVIII. Therefore, the surface frequency/density of this pVIII mutation (1 for every 200 nm^2^) is substantially lower than the one for the peptides displayed on the minor capsid (2–3 pIII for every 1 nm^2^). Thus, considering these structural differences, the assumption that pIII displayed peptides affinity to nanoparticles would be conserved when displayed on the pVIII may not always be accurate. Finally, given the challenge for reproducibly building functional structures at the nanometer scale, the streamlined methodology reported here may serve as a complementary approach to understand and to gain control of the assembly of biologically based nanoparticles with or without genetic manipulation of phage capsid. The modularity of phage and the number of possible applications for such bioinorganic hydrogels along with its versatility at various pH is a needed characteristic for the fabrication and implementation of economically relevant materials which can produce practical bio-electronic, environmental and biomedical tools.

## Materials and Methods

### Au-phage hydrogel assembly

The assembly of Au-phage hydrogel was achieved by adding Au NP (1.2 pM) in equal volumes to 150 µl aqueous solution of phage (native or mutant with light extinction of 0.14 a.u. at 270 nm) in aqueous solution of varying pHs (5 mM HCl pH 2, 5 mM Glycine buffer for pHs 2.5, 3.0, and 3.5, 5 mM acetate buffer for pHs 4.0 and 5.0, 5 mM boric acid for pH 5.5, 5 mM borate buffer for pH 8.5, 5 mM NaOH pH 14). Hydrogels were allowed to form overnight at room temperature. The 50±8 nm Au NP solution, verified by transmission electron microscope (TEM) image analysis, was prepared following the common citrate-reduction [20] procedure (molar ratio of 0.8∶1 of sodium citrate:Au(III) chloride). Au(III) chloride (99.99+%) was purchased (Sigma-Aldrich).

### Uv-vis spectroscopy

The Uv-vis spectroscopy measurements were performed with a plate reader spectrometer (Spectramax M5, Molecular Devices).

### SPR spectroscopy

A Biacore 3000 was used for SPR measurements of phage adsorption onto sodium citrate (0.3 mM) treated Au surfaces (Biacore Life Scieces). Phage solutions were prepared with buffers of respective pH (listed above). After each phage adsorption measurement (20 µl/min flow rate) the Au surface was treated with 50 mM NaOH, with picopure H_2_O and 0.3 mM sodium citrate. Finally, the buffer background signal was subtracted from sample data for each respective condition.

### Phage

Native or mutant phage were prepared by means of bacterial infection [4], [5]. The mutant phage was genetically modified [4], [5] to display a peptide extension of arbitrary sequence (Fig. 2). The peptide extension repeats were displayed once every 26 times pVIII peptides were displayed (∼104 out of 2,700 peptide total for the major capsid composition) [4], [5].
